# Elucidating the possible mechanism of action of some pathogen box compounds against *Leishmania donovani*

**DOI:** 10.1371/journal.pntd.0008188

**Published:** 2020-04-10

**Authors:** Wandayi Emmanuel Amlabu, Christine Achiaa Antwi, Gordon Awandare, Theresa Manful Gwira

**Affiliations:** 1 West African Centre for Cell Biology of Infectious Pathogens, University of Ghana, Legon, Accra, Ghana; 2 Department of Zoology, Faculty of Life Sciences, Ahmadu Bello University, Zaria, Nigeria; 3 Department of Biochemistry, Cell and Molecular Biology, University of Ghana, Legon, Accra, Ghana; Ohio State University, UNITED STATES

## Abstract

Leishmaniasis is one of the Neglected Tropical Diseases (NTDs) which is closely associated with poverty and has gained much relevance recently due to its opportunistic coinfection with HIV. It is a protozoan zoonotic disease transmitted by a dipteran *Phlebotomus*, *Lutzomyia*/ *Sergentomyia* sandfly; during blood meals on its vertebrate intermediate hosts. It is a four-faceted disease with its visceral form being more deadly if left untreated. It is endemic across the tropics and sub-tropical regions of the world. It can be considered the third most important NTD after malaria and lymphatic filariasis. Currently, there are numerous drawbacks on the fight against leishmaniasis which includes: non-availability of vaccines, limited availability of drugs, high cost of mainstay drugs and parasite resistance to current treatments. In this study, we screened the antileishmanial activity, selectivity, morphological alterations, cell cycle progression and apoptotic potentials of six Pathogen box compounds from Medicine for Malaria Venture (MMV) against *Leishmania donovani* promastigotes and amastigotes. From this study, five of the compounds showed great promise as lead chemotherapeutics based on their high selectivity against the *Leishmania donovani* parasite when tested against the murine mammalian macrophage RAW 264.7 cell line (with a therapeutic index ranging between 19–914 (promastigotes) and 1–453 (amastigotes)). The cell cycle progression showed growth arrest at the G0-G1 phase of mitotic division, with an indication of apoptosis induced by two (2) of the pathogen box compounds tested. Our findings present useful information on the therapeutic potential of these compounds in leishmaniasis. We recommend further *in vivo* studies on these compounds to substantiate observations made in the *in vitro* study.

## Author summary

There are numerous drawbacks in the fight against leishmaniasis which includes difficulty in drug administration, lengthy time of treatment, high toxicity, adverse side effects, high cost of drugs and increasing parasite resistance to treatment. These have made the search for new antileishmanial chemotherapeutics very essential. The Medicine for Malaria Venture (MMV) with the aim of accelerating drug development for poverty-related diseases has assembled some 400 diverse, drug-like molecules active against neglected diseases called the Pathogen box compounds. Thus, in this study we explored the antileishmanial potency and elucidated some possible mechanisms of action of some of the compounds against the *Leishmania donovani* parasites. The six compounds studied caused a distortion in the mitochondrion morphology, loss of kinetoplastid DNA and eventual nuclear degeneration upon treatment for 72 hours. Parasites treated with two of the cytocidal compounds MMV676057 (E03C) and MMV688942 (D06A) showed no significant programmed cell death due to apoptosis when compared to the untreated parasites but rather showed a cell cycle growth arrest in the G0-G1 and S-phases.

## Introduction

Leishmaniasis is one of the Neglected Tropical Diseases (NTDs) whose global epidemiological distribution is unequaled due to many underlying factors. These includes the mass human migration due to civil unrests by wars, which has been responsible for the disease recrudescence in areas where its morbidity was once under control, differential ecological requirements of active vector species that affects transmission epizoology, availability of accessible and active reservoir hosts etc. Leishmaniasis is a zoonotic disease of the tropics and subtropical regions of the world that is associated with poverty and it is gradually gaining grounds in its spread due to its opportunistic infection with HIV [1, 2].

*Leishmania* species are the causative agents of these four faceted diseases (cutaneous, diffuse cutaneous, visceral and mucocutaneous leishmaniasis). They are obligate parasites that resides intracellularly in the vertebrate host’s macrophages and the visceral organs. They are transmitted to the vertebrate hosts through the bite of a dipteran vector, known as *Phlebotomus* sandfly or *Lutzomyia*. This protozoan parasite belongs to the Family: Trypanosomatidae and the Class: Kinetoplastida. Its lifecycle involves a sexual reproductive phase in the vector sandfly (definitive host) and an asexual phase in the vertebrate intermediate host. Leishmaniasis is pathologically presented as a cutaneous, diffuse cutaneous, visceral and mucocutaneous form in humans; with the visceral leishmaniasis being the deadliest when left untreated. It is a disease with much stigmatization due to the scars and deformation it leaves behind after healing (especially in the case of the cutaneous and mucocutaneous leishmaniasis) [3]. The Cutaneous leishmaniasis is the most common around the world and could be self-healing after some months and even years. Most epidemiological data obtained on this disease are insufficient due to its under-reportage owing to the fact that its proper diagnosis is poor in most endemic areas and so, it is usually mistaken for other diseases such as leprosy. However, recently, more cases of the disease are being reported from non-transmission active regions in humans, dogs and cats; where the cat is a new reservoir host and should not be neglected as a potential vehicle in this disease transmission dynamics [4–6].

Currently, there is no viable vaccine against leishmaniasis and so, disease control is largely through chemotherapy and possible vector control; either by avoiding the sand fly bites or the use of insecticides to repel/ kill the flies. However, the mainstay drugs against leishmaniasis are challenged by some limitations which include, toxicity against non-target cells, not being readily available to patients, long treatment regimen which discourages the drug intake by the patients, high cost (owing to the fact that most of the patients are poor) and the issue of drug resistance being reported in some endemic areas [1, 3, 7]. Thus, the need for new antileishmanials with better promise in terms of high selectivity, affordability and accessibility cannot be overemphasized [2, 4, 8].

In this study we tested six Pathogen Box Medicine for Malaria Venture (MMV) compounds for their antileishmanial activity *in vitro* against *Leishmania donovani* promastigotes and amastigotes. In addition, their possible mode of actions and selectivity against the parasite were also evaluated in a bid to show their tenability as possible leads for new therapeutics against leishmaniasis.

## Materials and methods

### Compounds

Six Pathogen box compounds were tested in this study and their identities are; MMV676057 (E03C), MMV688942 (D06A), MMV188296 (G04A), MMV688776 (B05A), MMV688934 (B06A) and MMV202553 (F05A). Stock solutions of 10 mM in 100% DMSO of each was obtained from the Pathogen box and a working solution for each was formulated with double distilled water, with the final DMSO concentration being at less than 1%. The positive control used is an established antileishmanial drug, amphotericin B (Sigma Aldrich, USA), which was reconstituted in double distilled water.

### *In vitro* cultivation of *Leishmania donovani* and RAW macrophage cell line

*Leishmania donovani* (MHOM/SD/62/1S strain) was a kind gift from Dr. Yamthe Lauve (Bei Resources NIAID, NIH). The promastigotes were cultured in M199 medium supplemented with 10% heat-inactivated fetal bovine serum (FBS), penicillin G sodium (100 μg/mL) and streptomycin sulfate (100 μg/mL) at 25° C and subcultured every 72 hours in the same medium at a mean density of 1 x 10^5^ cells/mL [9]. The murine macrophage cell line RAW 264.7 (RIKEN BioResource Centre Cell Bank, Japan) were kindly provided by Professor Regina Appiah-Opong of the Clinical Pathology Department, Noguchi Memorial Institute for Medical Research, Ghana. RAW 264.7 were grown at 37° C in DMEM medium (pH 7.4) supplemented with 10% heat-inactivated FBS for 48–72 hours in 5% CO_2_ and subcultured in fresh DMEM medium at a mean density of 1 x 10^5^ cells/mL.

### *In vitro* antileishmanial activity assay for both promastigotes and macrophage-amastigotes model

To determine the IC_50_, i.e., the concentration of the compound that inhibits growth of 50% population of the *Leishmania* parasites; promastigotes at a density of 2 x 10^4^ cells were incubated without or with the compounds in triplicates, at varying concentrations (0.0156–0.25 μg/mL) and kept for 4 days at 25° C. Amphotericin B was used as a reference antileishmanial drug (positive control). RAW 264.7 macrophage cells were infected with promastigotes at a density of 1:10 ratio for 12 hours, after which the non-phagocytosed promastigotes were discarded (by aspirating them alongside the media) and the amastigote cells within the macrophages were treated with the compounds (in fresh complete media) to test their growth inhibitory activity using the method described previously [10]. Parasite viability was assessed using the 3-(4,5-Dimethylthiazol-2-yl)-2,5-diphenyltetrazolium bromide (MTT) method in which the rate of formazan crystal formation was used as an index of cell viability as described by Mosmann [11]. Fluorescence was measured with a Varioskan Lux Elisa microplate reader (Thermo Fischer Scientific, USA) at a wavelength of 570 nm. The fluorescence counts were plotted against the drug concentrations and the 50% inhibitory concentration (IC_50_) was determined by analysis of the dose-response curves using excel [9, 12, 13].

### Cytotoxicity test against RAW macrophage cell line using MTT assay

A murine macrophage cell line (RAW 264.7) was cultured in DMEM medium supplemented with penicillin G sodium (100 μg/mL), streptomycin sulfate (100 μg/mL) and 10% FBS. Macrophages at a density of 1 x 10^6^ cells/mL were incubated at different concentrations (ranging from 0.39–100 μg/mL) of the compounds for 48 hours in a CO_2_ incubator (5% CO_2_, 37° C). Macrophages without any treatment were taken as negative control and amphotericin B was used as positive control. At the termination of the assay, MTT was added and incubated for an additional 3 hours. Acidified isopropanol was added to the set-up to solubilize the formazan crystals formed by the viable cells. The intensity of formazan formation was read on an Elisa plate reader (Varioskan Lux Elisa microplate reader, Thermo Fischer Scientific, USA) at 570 nm. The 50% cytotoxic concentrations (CC_50_) of the compounds were determined by analysis of dose-response curves. Selectivity index or therapeutic index was calculated as a ratio of CC_50_ RAW 264.7/IC_50_ *L*. *donovani* promastigotes or amastigotes [11, 12].

### Haemolysis assay against the human red blood cells

The haemolytic effect of the compounds were tested against the human red blood cells at a density of 2% haematocrit. The method described by Kazi *et al* [14] was used with slight modification. In this study, human red blood cells were used, the respective test compounds were incubated for 3 hours and 1% triton X100 served as the positive control. At the end of the assay the supernatant was read on a microtiter plate reader (Varioskan Lux Elisa microplate reader, Thermo Fischer Scientific, USA) at 415 nm. The 50% haemolytic concentration (HC_50_) of the compounds were determined by analysis of dose-response curves.

### Growth kinetics and growth reversibility assays against the promastigotes of *Leishmania donovani*

Promastigotes of *L*. *donovani* (2 x 10^4^ cells) were incubated at 25° C in the presence of the test compounds (at 1μg/mL for each compound) in M199 containing 10% FBS (complete medium). Promastigotes treated with amphotericin B (0–50 μg/ml) served as positive control while untreated promastigotes in the medium alone served as negative control. Aliquots from treated and control parasites were diluted in 0.02 M PBS (pH 7.2) and the viable cells counted daily for 5 days using a Neubauer haemocytometer and a phase-contrast microscope under a 40x objective [9, 15].

However, to confirm the cytocidal or cytostatic effect of the test compounds, the treated and untreated parasites after 5 days of incubation were washed twice with fresh M199 and then resuspended in complete M199 media and allowed to grow further at 25° C; the viable promastigotes were enumerated microscopically after 4 days [9].

### Analysis of morphological and mitochondrion integrity of *Leishmania donovani* promastigotes using fluorescence microscopy

*Leishmania donovani* promastigotes (2 x 10^4^ cells) were treated with the test compounds at 1μg/mL (point concentration). They were then incubated for 24 hours and 72 hours in order to study their morphological changes and to see the changes in their mitochondrial integrity using DAPI and MitoTracker red dye respectively. The assay was performed using the methods described by Amisigo *et al* [16] with slight modifications. Here the Mitotracker red dye was added to the pelleted promastigote cells at a concentration of 100 nM, resuspended in 1 mL of FBS-free M199 media with 1% BSA (Bovine Serum Albumin) and incubated for 30 minutes.

### Cell cycle progression analysis of promastigotes treated with the MMV compounds

*Leishmania donovani* promastigotes (2 x 10^4^ cells) were treated at 2x IC_50_ concentrations of the respective test compounds and compared against a negative control of untreated promastigote cells in a 24 hours incubation period. Guava Cell Cycle Reagent (catalog No. 4500–0220) was used to stain the cells and the number of cells in G0/G1, S and G2/M phases were determined [17]. Briefly, treated and untreated cells were pelleted and fixed in 70% ice-cold ethanol for an hour at -20°C, the suspended cells were then pelleted by centrifugation at 1500 rpm for 10 minutes. The supernatants were discarded, and the pelleted cells were washed twice with 1X PBS to ensure total removal of the ethanol. To a 200 μL of the cell pellets, a 200 μL of the Gauva Cell Cycle Reagent was added and incubated at room temperature (25°C) for 30 minutes in the dark. Cell distribution at the distinct cell cycle phases were measured using flow cytometer (BD LSR Fortessa X-20 analyzer BD Biosciences San Jose, CA 95131 USA). Data generated were analyzed using the FlowJo V10 software.

### Apoptotic and necrotic potentials of test compounds against the promastigotes of *Leishmania donovani*

Promastigotes of *Leishmania donovani* (2 x 10^4^ cells) were treated at 2x IC_50_ concentrations of the respective test compounds against a negative control of untreated promastigote cells in a 24 hours incubation period and the cells were prepared using the Annexin V-FITC apoptosis detection kit following the Sigma protocol (Catalog Number: APOAF). Briefly, treated promastigote cells were washed twice with cold 1x PBS and then resuspended in 1x binding buffer at a cell density of 1 x 10^6^ cells/mL. To a 100 μL of the suspended cells, 5 μL of Annexin V-FITC and 5 μL of Propidium Iodide (PI) were added. The mixture was gently vortexed and allowed to incubate for 15 minutes at room temperature (25°C) in the dark. To the set-up, 400 μL of 1x binding buffer was added and the assay was read by flow cytometry (BD LSR Fortessa X-20 analyzer BD Biosciences, San Jose, CA 95131 USA) and the results analyzed using FlowJo version 10 software.

### Statistical analysis

The cell viability studies with results presented as IC_50_, CC_50_ and HC_50_ were obtained from analysis of dose-response curves using excel and the data expressed as mean ±standard deviation (SD) of three independent experiments. ANOVA was used to determine the statistical significance (P<0.05) between treatment groups in the growth kinetic and reversibility studies using the Graph Pad Prism 6.0 Software. Cell cycle and apoptosis studies were done by flow cytometry and the results analyzed using FlowJo V10 software. Student t-test was used to establish their statistical significance at P<0.05.

## Results

### Antileishmanial, cytotoxicity and haemolysis profile of compounds against *Leishmania donovani*

The antileishmanial activity of six pathogen Box MMV compounds (MMV676057 (E03C), MMV202553 (F05A), MMV188296 (G04A), MMV688776 (B05A), MMV688934 (B06A) and MMV688942 (D06A)) were established both in the promastigote and amastigote forms of the *Leishmania donovani* parasite *in vitro* (S1 Fig and S2 Fig). These compounds were selected because they are among the 68 out of the 400 compounds in the pathogen box known to be kinetoplastid sensitive. All the six compounds were potent against both forms of the parasite with MMV688776 (B05A) being the most effective and MMV688942 (D06A) being the least effective against promastigotes, while MMV688934 (B06A) was the most effective and MMV688942 (D06A) being the least effective against amastigotes (Table 1). The cytotoxicity of the compounds was verified using a murine macrophage cell line RAW 264.7 (S3 Fig), in which a therapeutic index range of 1–914 was observed for both forms of the parasite (Table 1). Five of the compounds showed a good level of selectivity on the parasite. Haemolysis assay was conducted on all the compounds to ascertain their safety against the human red blood cells, and none of the compounds showed haemolytic effect against the human red blood cells (at least to the highest concentration tested).

**Table 1:** The 50% inhibitory concentrations (IC_50_) of MMV compounds, their haemolytic profile (HC_50_) and selectivity against the promastigotes and amastigotes of *Leishmania donovani*.

Compound code	MMV compound ID	IC_50_ (μM)±SD promastigotes	TC_50_(μM)±SD RAW	Therapeutic Index (TC_50_/IC_50_) promastigotes	IC_50_(μM)±SD amastigotes	Therapeutic Index (TC_50_/IC_50_) amastigotes	HC_50_ (μg/mL)
E03C	MMV676057	22 ± 0.04	9512 ± 0.05	432	594 **±** 0.01	16	>100
F05A	MMV202553	51 ± 0.02	14853± 0.10	291	43 **±** 0.009	345	>100
G04A	MMV188296	14 ± 0.01	12806 ± 0.06	914	54 **±** 0.007	237	>100
B05A	MMV688776	2.6 ± 0.01	50 ± 0.02	19	38 **±** 0.03	1	>100
B06A	MMV688934	25 ± 0.06	10420 ± 0.01	416	23 **±** 0.006	453	>100
D06A	MMV688942	273 ± 0.12	11855 ± 0.01	43	740 **±** 0.005	16	>100
Amphotericin B		5.3 ± 0.03	15.75±0.03	3.0	ND	ND	ND

ND = not determined, TC_50_ = Toxic concentration against 50% of cell population, HC_50_ = Haemolytic concentration against 50% of cell population.

### The effect of the MMV compounds on the growth kinetics of *Leishmania donovani* promastigotes and their cytostatic/ cytocidal potentials

**Fig 1:**
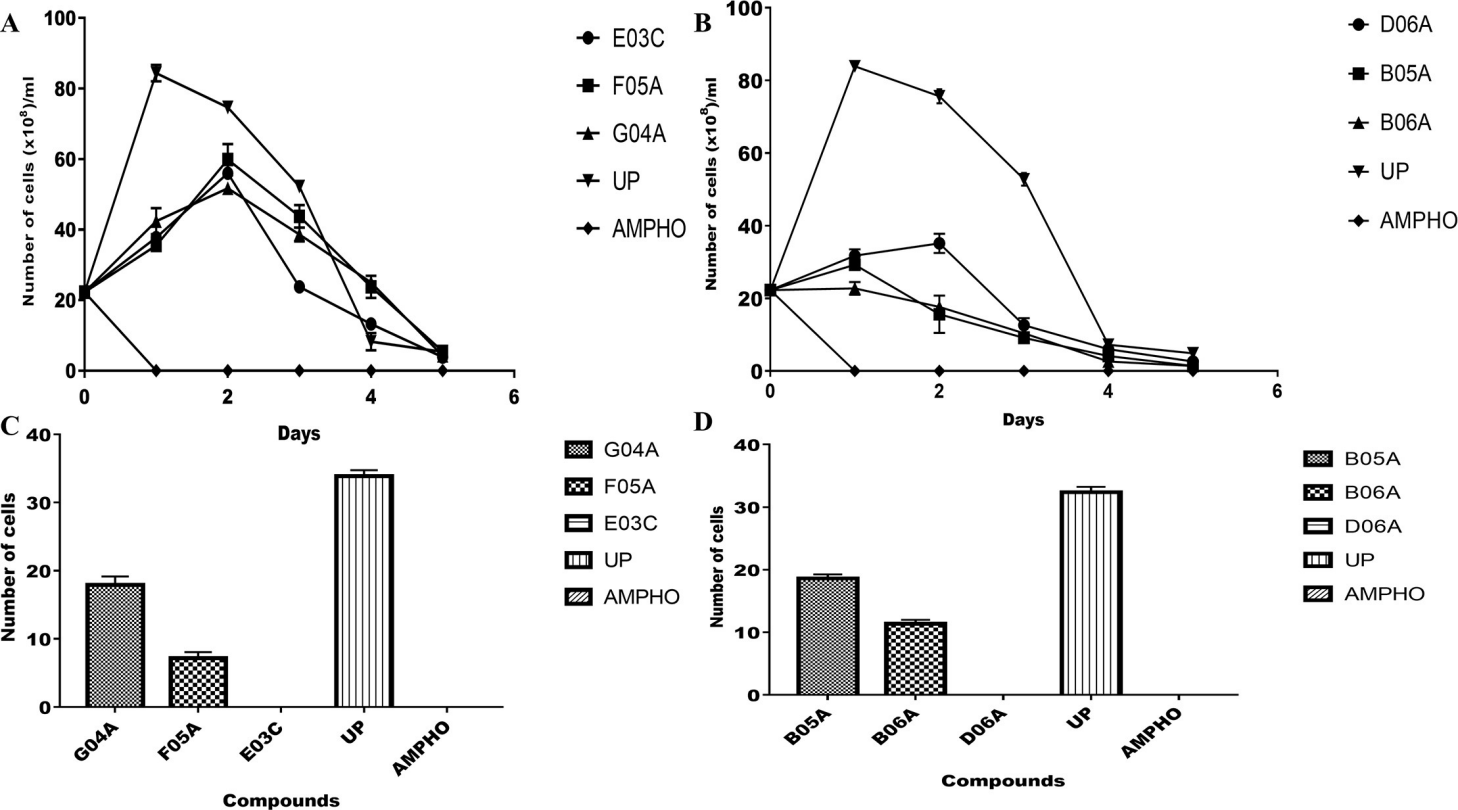
Growth kinetics, cytocidal and cytostatic effect of the compounds on *Leishmania donovani*. (A) The growth kinetics curves of MMV676057 (E03C), MMV202553 (F05A), and MMV188296 (G04A) compounds against *Leishmania donovani* promastigotes enumerated within 5 days of incubation. Here, 1μg/ml of the compounds were used. (B) The growth kinetics curves of MMV688776 (B05A), MMV688934 (B06A), and MMV688942 (D06A) compounds against *Leishmania donovani* promastigotes enumerated within 5 days of incubation. Again, 1μg/ml of the compounds were used. (C) The growth reversibility profile of the compounds MMV676057 (E03C), MMV202553 (F05A), and MMV188296 (G04A). (D) The growth reversibility pattern of the compounds MMV688942 (D06A), MMV688776 (B05A) and MMV688934 (B06A). UP = untreated parasites and AMPHO = amphotericin B, the positive control). All data shows the mean of three independent experiments. In Fig 1A and 1B, ANOVA was used to analyze the significant differences between treatments at P<0.05.

The growth kinetic profile of the *Leishmania donovani* promastigotes were monitored after treatment with the six compounds in order to determine the pattern at which parasite populations diminishes in the presence of the test compounds within the period of 120 hours compared with the negative control. The growth curve indicated that the compounds were inhibiting growth of the parasites when compared with the untreated control (Fig 1A and 1B). The growth reversibility profile of the compounds were examined to investigate their cytocidal/ cytostatic potentials (Fig 1C and 1D), The growth inhibition of parasites treated with MMV676057 (E03C) and MMV688942 (D06A) were not reversible while the promastigotes treated with the remaining four (4) compounds (MMV188296 (G04A), MMV688776 (B05A), MMV688934 (B06A) and MMV202553 (F05A)) showed 56.5%, 57.9%, 32.3% and 19.5% growth reversal respectively (Fig 1C and 1D).

### The effects of MMV compounds on the morphology and mitochondrion integrity of *Leishmania donovani* promastigotes

**Fig 2:**
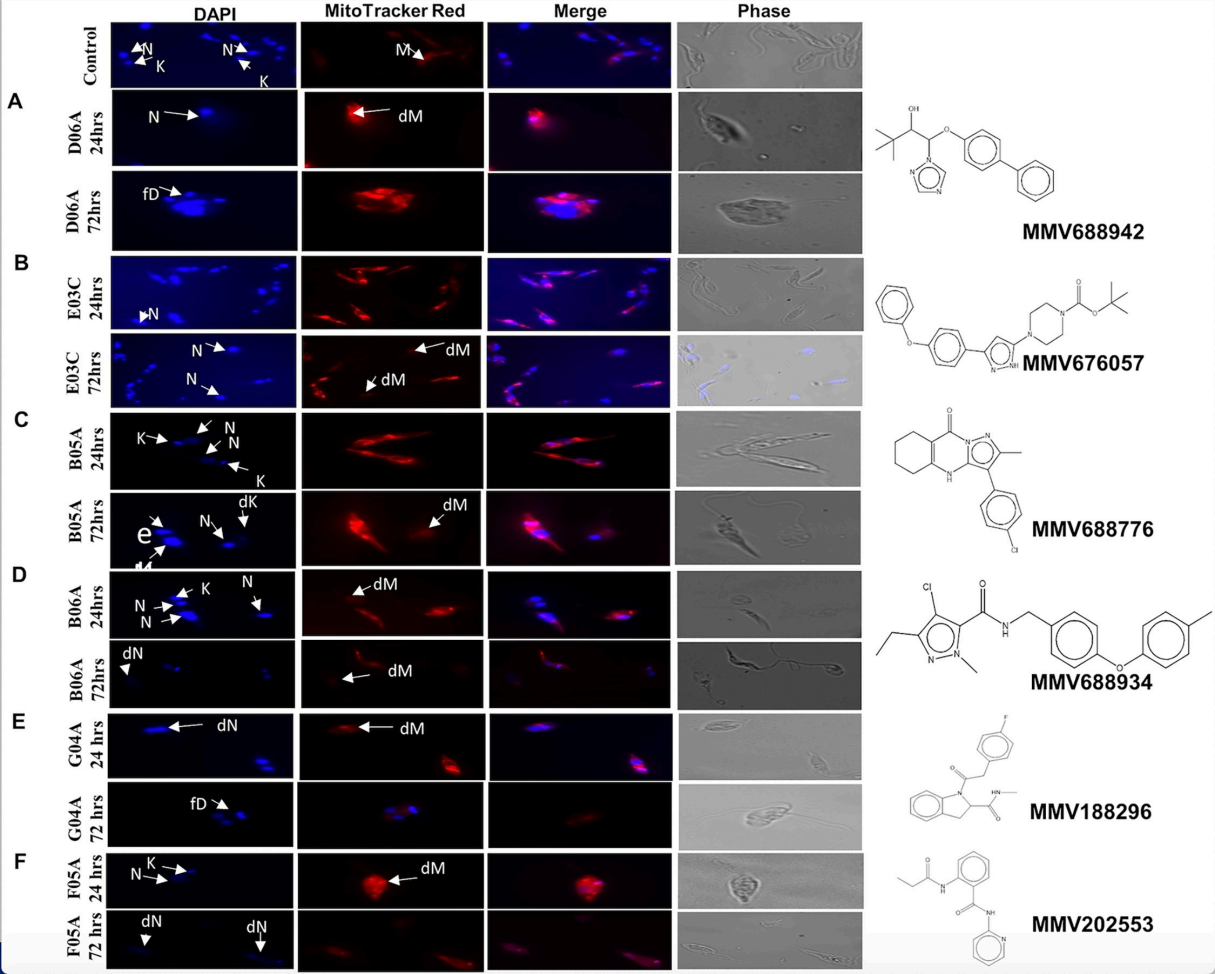
Effect of compounds on the nuclear and kinetoplast DNA, and mitochondria morphology. (A) MMV688942 (D06A) (B) MMV676057 (E03C) (C) MMV688776 (B05A) (D) MMV688934 (B06A) (E) MMV188296 (G04A) (F) MMV202553 (F05A). Promastigotes were cultured in the presence of MMV compounds for 24 hours and 72 hours, stained with DAPI and Mitotracker, and examined by phase contrast microscopy and fluorescence microscopy at x1000. Here, 1μg/ml of the compounds were used. Key: fD = Fragmented DNA; dM = Degenerated Mitochondrion; M = Normal Mitochondrion; N = Nucleus; K = Kinetoplast; eK = enlarged Kinetoplast; dN = Degenerated Nuclei DNA; fD = Fragmented DNA.

We examined the morphology and mitochondrion integrity of the parasites and observed that the promastigotes upon treatment with the compounds after a 24 and 72 hours incubation period showed abnormalities in their sizes and shape relative to the untreated controls (Fig 2A–2F). Promastigotes treated with MMV688942 (D06A) showed loss of kinetoplast in about 30% of the parasites after 24 hours treatment, however after 72 hours, there was an abnormal disintegration of the DNA and mitochondrion in 85% of the parasite cells observed (Fig 2A). About 70% of the parasite cells treated with MMV676057 (E03C) or MMV688776 (B05A) showed no obvious DNA and mitochondrion abnormality after 24 hours, however, there was an obvious degeneration of the DNA and mitochondrion in about 85% of the parasite treated with MMV676057 (E03C) (Fig 2B) and almost 70% of the cells treated with MMV688776 (B05A) after 72 hours treatment (Fig 2C). In addition, parasites treated with MMV688776 (B05A) for 72 hours were observed having their kinetoplast and nuclei DNA being disproportionately enlarged (Fig 2C). Further, 70% of MMV688934 (B06A) treated promastigotes showed enlargement of the nucleus and kinetoplast with the mitochondria losing its normal shape after the 24 hours treatment. However, after 72 hours there was degeneration of the mitochondria, nucleus and kinetoplast DNA in more than 90% of the cells (Fig 2D). The cells showed a loss in mitochondrion integrity in 40% and 60% of the parasite population after 24 hours treatment with MMV188296 (G04A) and MMV202553 (F05A) respectively. However, after 72 hours there was degeneration of the nucleus and kinetoplast DNA, and the mitochondria structure in more than 90% of the parasite cells (Fig 2E and 2F).

### Cell cycle progression of promastigotes treated with MMV676057 (E03C) and MMV688942 (D06A) compounds.

**Fig 3:**
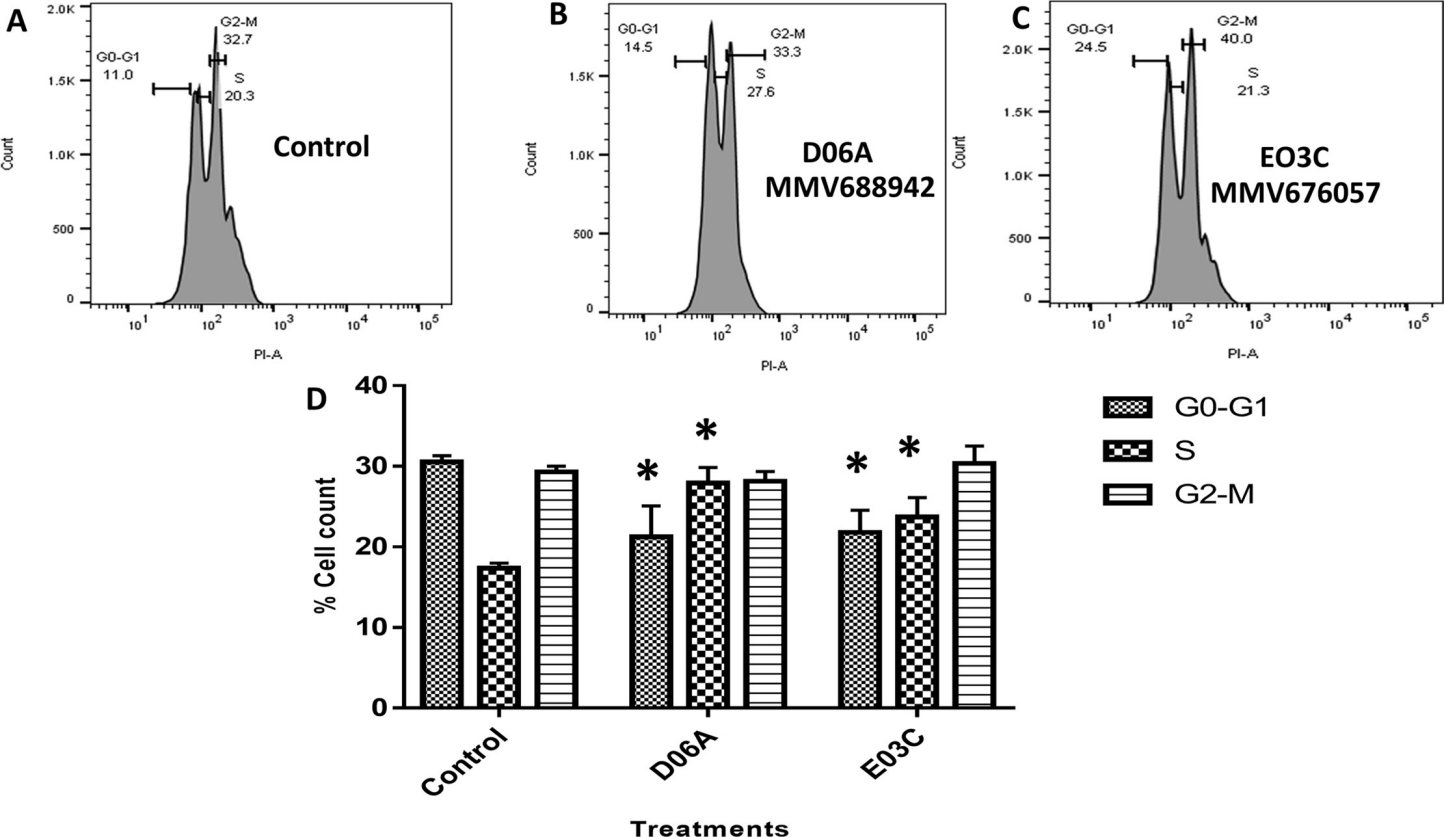
Cell cycle progression of *Leishmania donovani* treated cells. Flow cytometry histograms of (A) Untreated (B) MMV688942 (D06A) (2x IC_50_). (C) MMV676057 (E03C) (2x IC_50_). Parasites were cultured with compound for 24 hours. Untreated promastigotes cells were used as negative control. (D) The percentage quantification of cells at each cell cycle phase. Cell counts in treated wells were compared to those in untreated wells using Student’s t test, and statistical significance was set at *P<0*.*05*. Data shown are representative of three independent experiments.

To determine the effects of treatment with the MMV compounds on the normal cell cycle progression of *Leishmania donovani*, the DNA content of treated and untreated cells incubated for 24 hours were analyzed by flow cytometry (Fig 3A–3C). Analysis of the cell cycle progression of the promastigotes showed a reduction in the number of cells at the G0-G1 phase when treated with MMV688942 (DO6A) (22%, P value = 0.0001) and MMV676057 (E03C) (23%, P value = 0.0001) compared to the untreated parasite control (untreated 30%). However, there was significant increase in the population of cells at the S phase (MMV688942 (DO6A) = 28%, P value = 0.0002 and MMV676057 (E03C) = 23%, P value = 0.0002) with no significant change in the number of cells at the G2/M phase (Fig 3D).

### Apoptotic and necrotic potentials of the MMV compounds MMV676057 (E03C) and MMV688942 (D06A) against the promastigotes of *Leishmania donovani*

**Fig 4:**
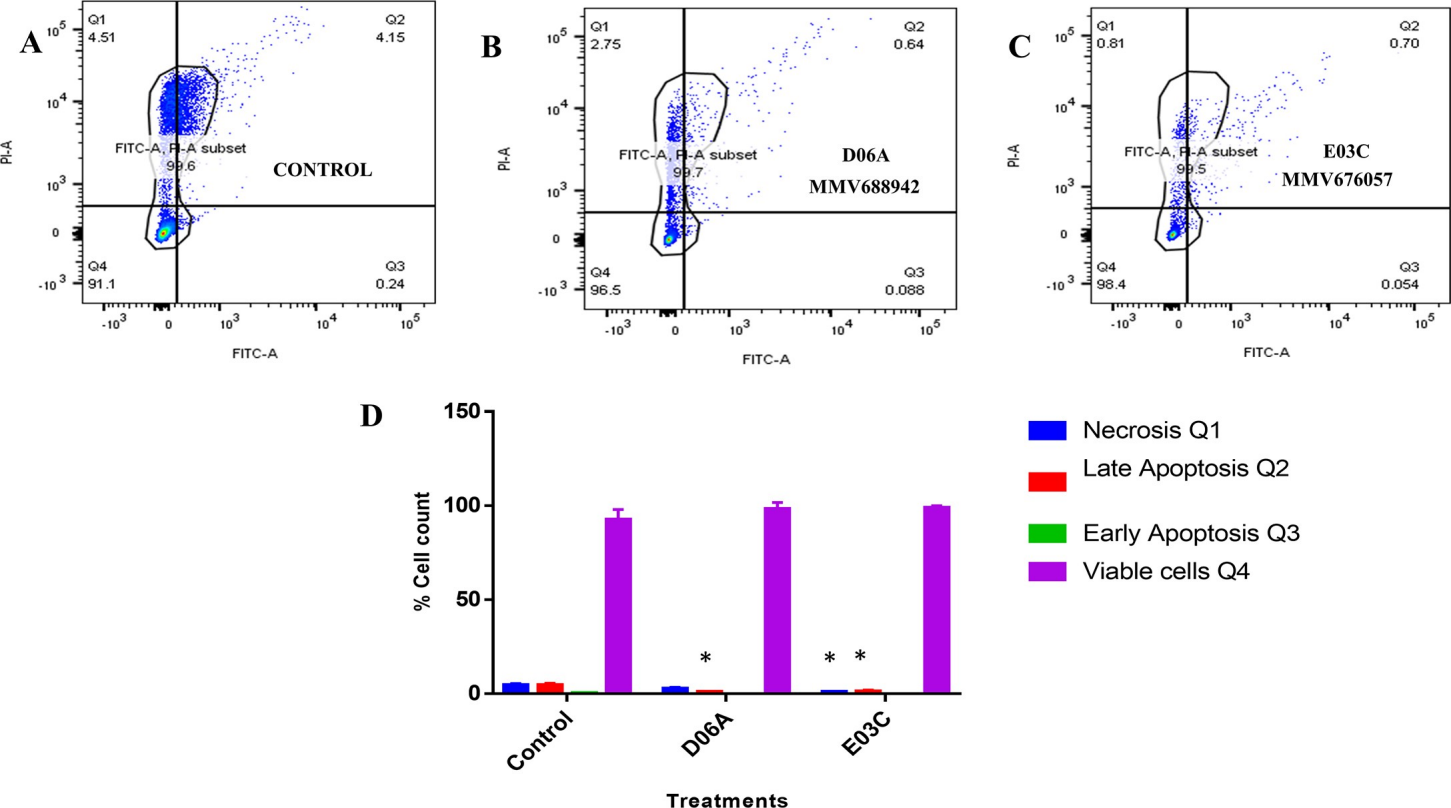
Effect of MMV688942 (D06A) and MMV676057 (E03C) compounds on promastigote cell death. (A) Untreated cells (negative control) and cells treated with (B) MMV688942 (D06A) (2x IC_50_) (C) MMV676057 (E03C) (2x IC_50_) (D) Percentage cell quantification of cells in the respective stages. Statistical significance was determined using unpaired t-test by comparing the treated and the control. These are representative profiles of three independent experiments.

The apoptotic potentials of the two compounds (MMV676057 (E03C) and MMV688942 (D06A)) were evaluated after 24 hours of incubation with the *Leishmania donovani* promastigote cells. The choice of a short course treatment regimen of 24 hours was to see if the compounds actually induce any apoptotic action on the parasite cells. Likewise, to deduce if the necrosis observed on the cells after a 72 hours treatment of cells with the compounds was likely due to an apoptotic inducement by the compounds at the earliest incubation time. The result obtained showed slight apoptosis and necrosis which was far less than what was observed in the untreated cells (Fig 4). The percentage of cells observed upon treatment with MMV688942 (D06A) and MMV676057 (E03C) in the necrotic, viable cells, late and early apoptosis stages compared with the negative control showed less counts in all the variables except in the number of viable cells, that were slightly higher (Fig 4A–4D).

## Discussion

The antileishmanial activity of six Pathogen Box MMV compounds were screened against the promastigote and amastigote stages of *Leishmania donovani* and IC_50s_ ranging from 2.6–273 μM and 23–740 μM were obtained respectively. Earlier report by Kaiser and colleagues [18] however recorded an IC_50_ of 1.3 and >10 μM for *Leishmania donovani* promastigote and amastigote respectively for MMV676057 compound and an IC_50_ of 13 μM for *L*. *major* and 10.2 μM for *L*. *infantum* against the same compound. Whereas, an IC_50_ range of 44 –>64 μM was reported against *L*. *infantum* alone for the remaining five compounds [18] (https://www.mmv.org›uploads›docs›mmv_open).

The therapeutic index of these six compounds were evaluated after their cytotoxicity was assessed against the murine mammalian RAW cell line, and one of the compounds MMV688776, showed a very poor selectivity (1) against the amastigote stage of the parasite. The remaining five compounds (MMV676057, MMV202553, MMV188296, MMV688934 and MMV688942) had good selectivity (16–923) against the parasite. The haemolytic profile of all the compounds showed no haemolysis against the human red blood cells within the limits of the highest concentration tested in this study (>100 μg/mL). Our data therefore indicates that these compounds do not have any obvious adverse effect *in vitro* against the non-target cells but rather showed selectivity against the *Leishmania donovani* parasite [12].

A growth kinetics profile of the compounds upon incubation with the promastigote stage of the parasite were assessed. There was an observed decline in the parasite population after 48 hours of incubation with four (4) of the compounds (MMV676057, MMV202553, MMV188296 and MMV688942) while the other two (2) compounds; MMV688776 and MMV688934 showed population decrease much earlier; after 24 hours of drug incubation. This indicates that each compound had a peculiar time frame for drug bioavailability within target cells which could cause a corresponding expression of its possible impact on the parasite viability. Thus, suggesting a time-dependent killing of the parasites upon incubation with the compounds. However, there were no viable cells observed after 24 hours of incubation with the positive control amphotericin B an established anti-leishmanial drug [9]. The growth reversibility profile showed MMV676057 and MMV688942 were cytocidal, while MMV202553, MMV188296, MMV688776 and MMV688934 were cytostatic. The cytocidal compounds showed no viable promastigote cells after drug withdrawal while the cytostatic compounds showed some revived cells at varying densities. Those compounds with cytocidal effect on the parasite hold more promise as future chemotherapeutics against Leishmaniasis, while the cytostatic compounds could best be used as combination therapies with other less bioactive anti-leishmanial regimens that are cytocidal [19]. Thus, considering the overall potency of the compounds and their varying selectivity against the parasites, further evaluation of the cytotoxicity using a combination of the compounds against the parasite may present better leads. Also, further focus on the amastigote stage of the parasite and *in vivo* studies will be essential since it is this stage of the parasite that causes pathogenesis in the vertebrate host.

Studies on the morphology of the parasite cells treated with these compounds revealed some alterations from the normal phenotype after 24 hours incubation but more prominent changes observe after the 72hours treatment. The DAPI (4′, 6-diamidino-2-phenylindole) stained cells revealed the nature of the nucleus and the kinetoplast, while the Mitotracker dye revealed the nature of the mitochondria upon treatment with these compounds. The morphological aberrations observed included loss of the kinetoplast DNA, shrinkage of the cytoplasm, degeneration of the mitochondria and eventual complete disintegration of the parasite cells. However, treatment with amphotericin B had been reported to show irregular shape, severe distortion in the cell membrane with a loss of the kinetoplast DNA (10). These abnormalities observed suggests the likely causes of the cell deaths induced by the compounds. Reports have shown the ability of some genetically modified trypanosome species lacking the kinetoplast DNA (dyskinetoplastids) to have the ability to survive and replicate. This information suggested that the kinetoplast DNA was dispensable in those parasite species since its absence could not deter the respirational capabilities of the affected parasite cells [20]. However, recent studies reveal that the dyskinetoplastic parasites could only survive due to some adaptational mutations they underwent swiftly [21]. Therefore, having a drug candidate that efficiently targets and disrupts the kinetoplast DNA could be very effective; as it would rapidly tamper with the biological efficiency of the parasite and could cause its eventual cell death. To effectively cause the death of the parasites, the drug must be constantly available in the target-cell’s micro-environment, hence, not giving the parasite the desired time needed to adjust to its new adaptational challenges. The survival of induced dyskinetoplastic trypanosomes is greatly affected by the absence of the kinetoplast DNA which encoded important mitochondria proteins [22]. In this study we have shown the possibility of the degeneration of the kinetoplast DNA as being one likely mechanism of action employed by these compounds which led to the killing of the parasites.

The parasites mitochondria exhibit a unique structure and they are functionally distinct from the mammalian mitochondria, thus, making this organelle an exceptional chemotherapeutical target [23]. The change in the shape of mitochondria and its membrane integrity can be a likely cause of death of the parasite [24] and the intact shape of the mitochondrion is essential for its normal function, which when compromised could result in the death of the parasite [24, 25]. In this study, cells treated with the compounds appeared lysed with the loss of kinetoplast and nuclear DNA, and mitochondria integrity. The alteration in the mitochondria structure/ shape may be another possible mechanism of the cause of parasites death by the compounds [26].

The biological effect of MMV676057 and MMV688942 compounds on the cell cycle progression of the promastigotes showed a drastic decline in the G0-G1 phase and a rise in the S phase of the parasite cell cycle compared to the untreated parasite control. The decline in the G0-G1 phase indicates that there were no obvious increase in the sizes of the cell organelles which is characteristic of that phase and which progresses into duplication of those organelles at the S phase—result in an increase in cell counts at that phase and then translates into an alignment of the duplicated organelles ready for their equal polarization at the G2 phase and eventual division at the M phase (cytokinesis). Given that these organelles duplicate once during the cell cycle, it has been established that the extrusion of the daughter flagellum in the promastigotes precedes the onset of mitosis, which in turn ends after kinetoplast segregation, and that significant remodeling of cell shape accompanies mitosis and cytokinesis [27, 28]. However, the cell cycle arrest observed at the G0-G1 phase could be responsible for the decrease in parasite population as observed even in the growth kinetic curves and we infer that there is a cell cycle arrest by those compounds at that phase of the cell cycle which resulted in the eventual hampered growth/non-proliferation of parasite cells [25]

The apoptotic potentials of MMV676057 and MMV688942 were evaluated after 24 hours of incubation with the *Leishmania donovani* promastigote cells. The choice of a short course treatment regimen of 24 hours was to see if the compounds actually induce any apoptotic action on the parasite cells, and to deduce if the necrosis observed on the cells after a 72 hours treatment of cells with the compounds was likely due to an apoptotic inducement by the compounds at the earliest incubation time. The possibility of cell death in *leishmania* due to apoptosis has been proven and it has been established as a possible mode of action against the *Leishmania donovani* parasites upon treatment with certain antileishmanial compounds [7]. The result obtained showed slight apoptosis trigger. Furthermore, the role of mitochondrion disruption in orchestrating apoptosis has been implicated and that the irreversible damage and dysfunction of this vital organelle would have disastrous consequences on the survival of the parasites [29, 30]. Thus, the obvious damage observed on the mitochondrion shape of the parasite cells upon treatment with these compounds could be responsible for the level of apoptosis observed at the earliest time of parasite incubation with the compounds [7].

In this study we have shown the *in vitro* antileishmanial activities and some likely mechanism of action of some MMV compounds, MMV676057 (E03C), MMV202553 (F05A), MMV188296 (G04A), MMV688776 (B05A), MMV688934 (B06A) and MMV688942 (D06A) on the promastigote stage of *Leishmania donovani*. We report their potential in disrupting vital internal organelles of the parasites which invariably leads to their death. The high selectivity of these compounds suggests their promise as possible new leads for future anti-leishmanials.

## Supporting information

S1 Fig Concentration-response curves for the promastigotes of *Leishmania donovani*. Antileishmanial activity of the six MMV compounds against the promastigote stage of the parasite monitored by MTT assay. All data shown are the representation of three independent experiments.
(TIF)

S2 Fig Concentration-response curves for the amastigotes of *Leishmania donovani*. Antileishmanial activity of the six MMV compounds against the amastigote stage of the parasite monitored by MTT assay. All data shown are the representation of three independent experiments.
(TIF)

S3 Fig Concentration-response curves for the RAW 264.7 macrophage cell line. Cytotoxicity profile of the six MMV compounds tested against the RAW 264.7 macrophage cell line using the MTT assay. All data shown are the representation of three independent experiments.
(TIF)
