# Supplementary figures and images for: Elucidating the possible mechanism of action of some pathogen box compounds against Leishmania donovani
Source: PLoS Negl Trop Dis. 2020 Apr 10;14(4):e0008188. doi: 10.1371/journal.pntd.0008188 (PMC7176276; doi:10.1371/journal.pntd.0008188)

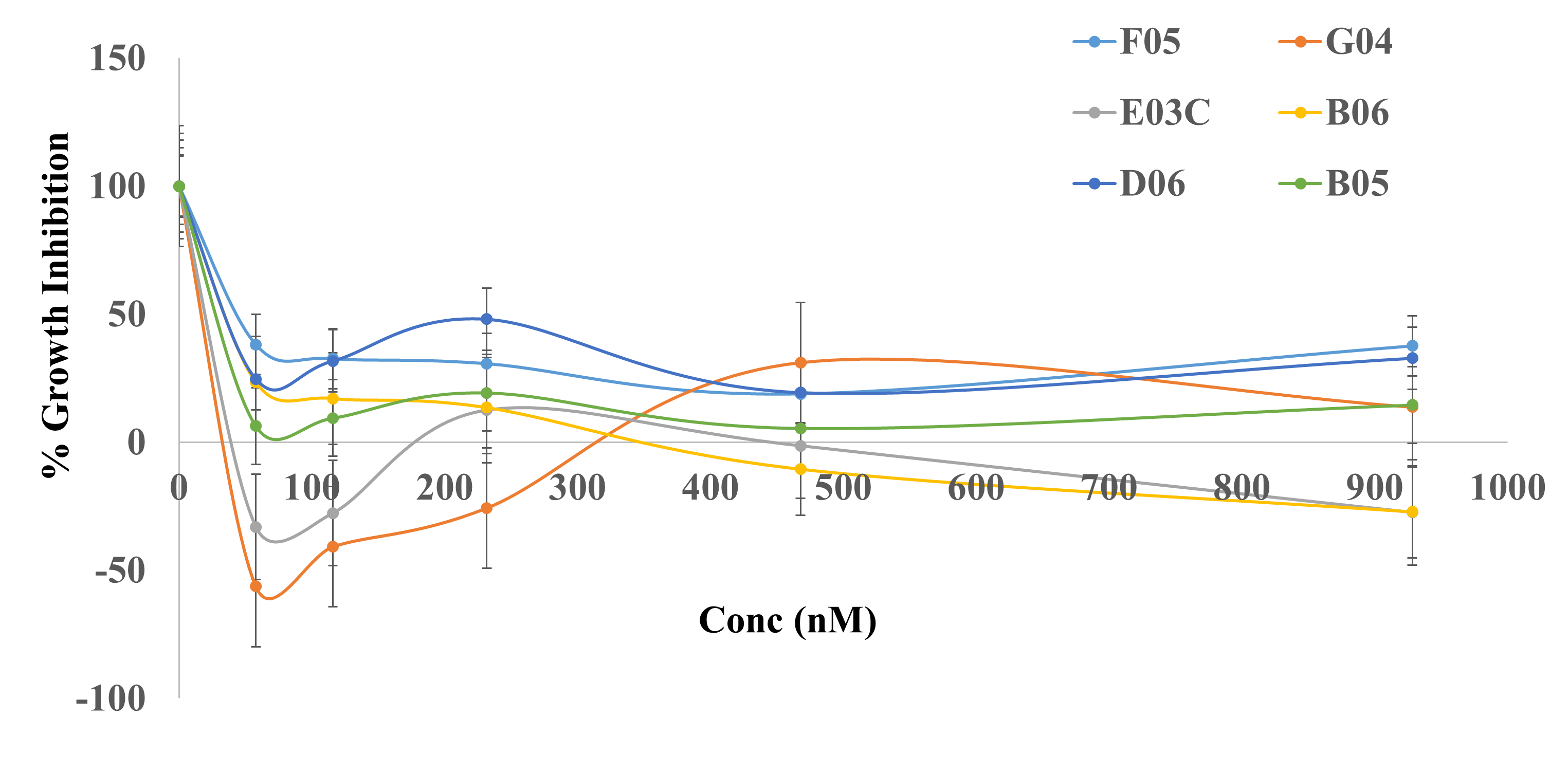

Supplement: S1 Fig — Antileishmanial activity of the six MMV compounds against the promastigote stage of the parasite monitored by MTT assay. All data shown are the representation of three independent experiments. (TIF) [file pntd.0008188.s001.tif]

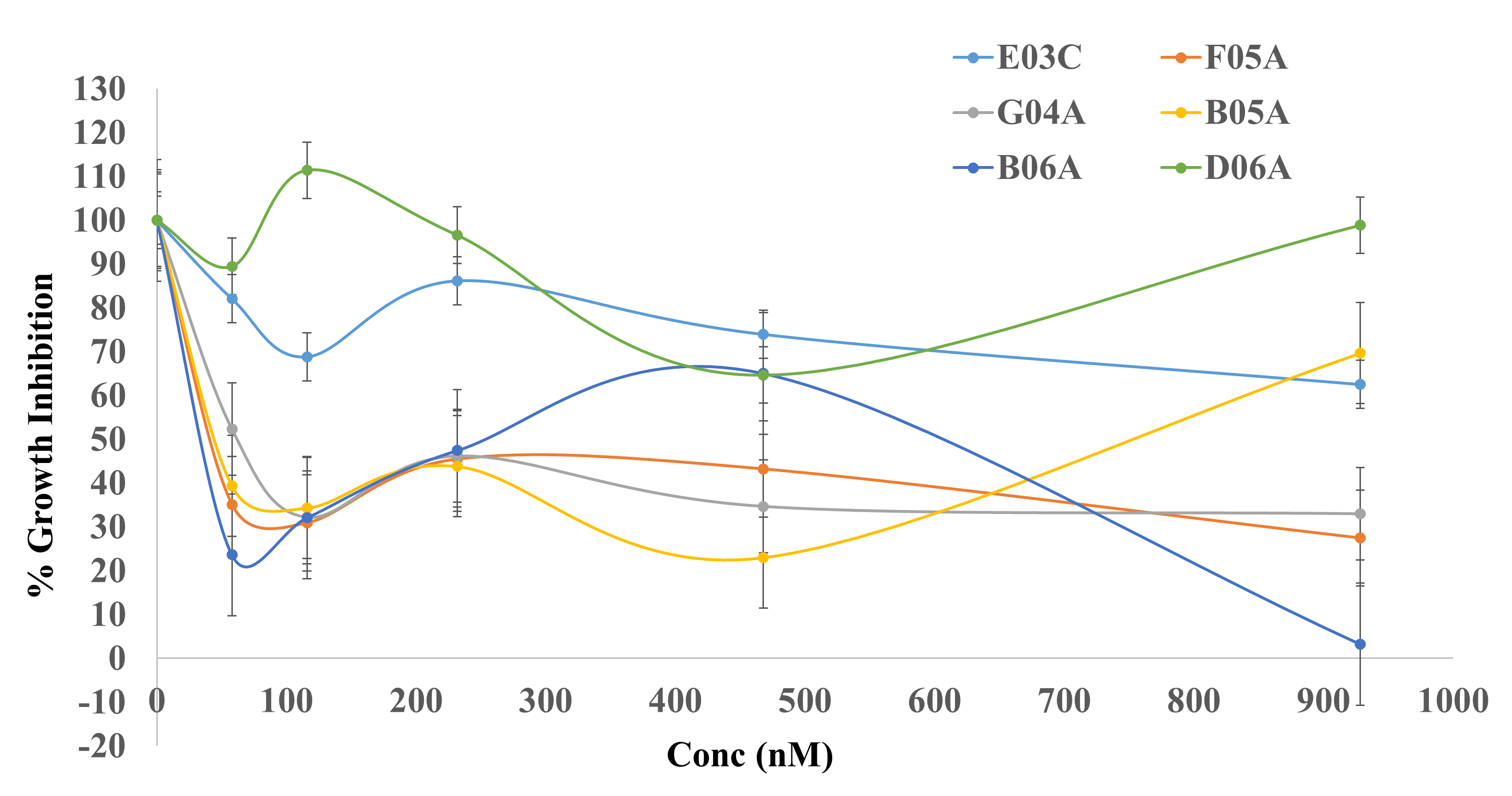

Supplement: S2 Fig — Antileishmanial activity of the six MMV compounds against the amastigote stage of the parasite monitored by MTT assay. All data shown are the representation of three independent experiments. (TIF) [file pntd.0008188.s002.tif]

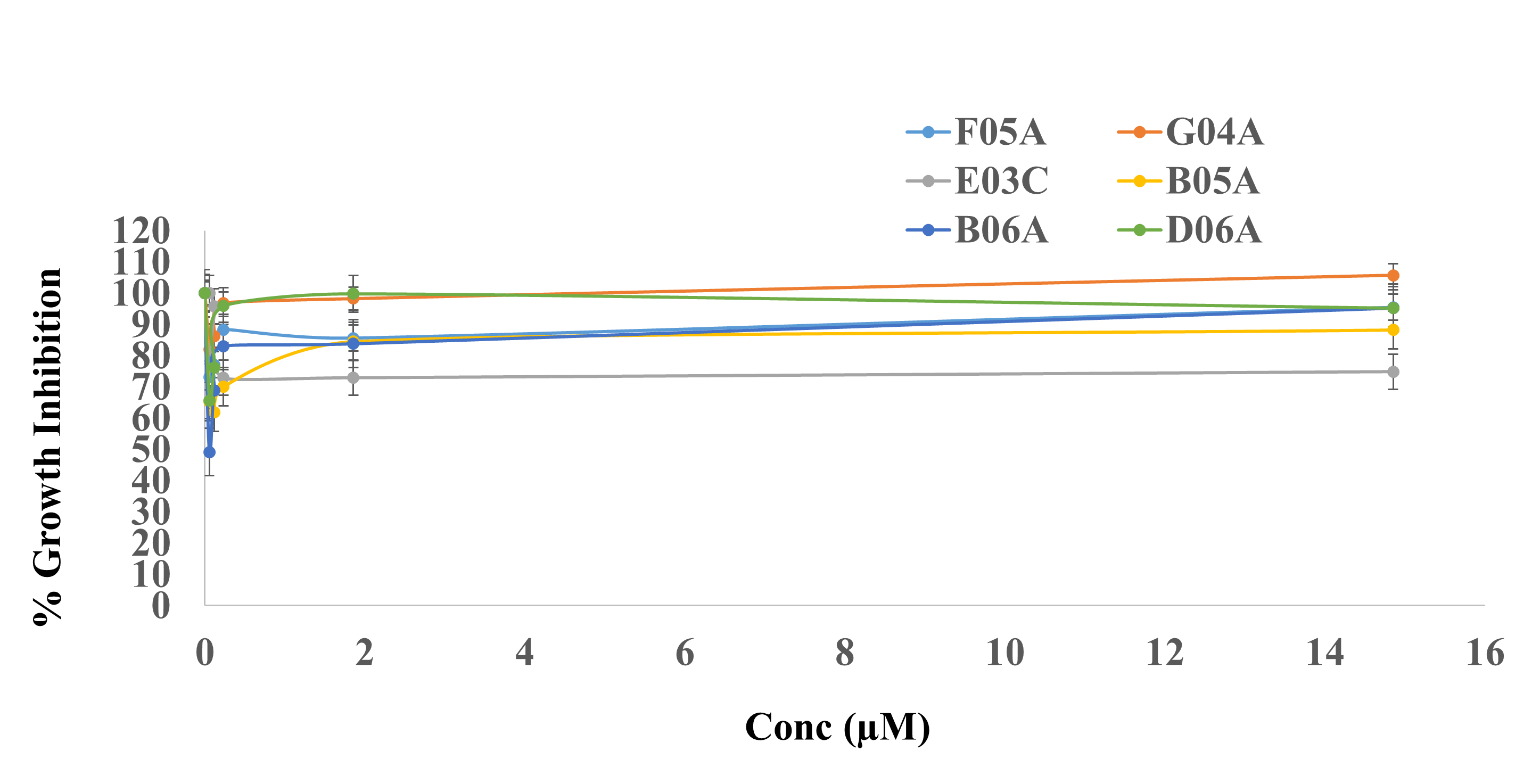

Supplement: S3 Fig — Cytotoxicity profile of the six MMV compounds tested against the RAW 264.7 macrophage cell line using the MTT assay. All data shown are the representation of three independent experiments. (TIF) [file pntd.0008188.s003.tif]
